# Performance Evaluation and Comparative Analysis of SubCarrier Modulation Wake-up Radio Systems for Energy-Efficient Wireless Sensor Networks

**DOI:** 10.3390/s140100022

**Published:** 2013-12-19

**Authors:** Joaquim Oller, Ilker Demirkol, Jordi Casademont, Josep Paradells, Gerd Ulrich Gamm, Leonhard Reindl

**Affiliations:** 1 Department of Telematics Engineering, Universitat Politècnica de Catalunya, C/Jordi Girona 1-3, Barcelona 08034, Spain; E-Mails: joaquim.oller@entel.upc.edu (J.O.); jordi.casademont@entel.upc.edu (J.C.); josep.paradells@entel.upc.edu (J.P.); 2 Fundació i2Cat, C/Gran Capità 2, Barcelona 08034, Spain; 3 Institut für Mikrosystemtechnik—IMTEK, Albert-Ludwigs-Universität Freiburg, Georges-Köhler-Allee 103, Freiburg 79110, Germany; E-Mails: gerd.ulrich.gamm@imtek.uni-freiburg.de (G.U.G.); reindl@imtek.uni-freiburg.de (L.R.)

**Keywords:** wake-up radio, wake-up receiver, wireless sensor networks, low-power communication, energy-efficient networking, performance analysis

## Abstract

Energy-efficient communication is one of the main concerns of wireless sensor networks nowadays. A commonly employed approach for achieving energy efficiency has been the use of duty-cycled operation of the radio, where the node's transceiver is turned off and on regularly, listening to the radio channel for possible incoming communication during its on-state. Nonetheless, such a paradigm performs poorly for scenarios of low or bursty traffic because of unnecessary activations of the radio transceiver. As an alternative technology, Wake-up Radio (WuR) systems present a promising energy-efficient network operation, where target devices are only activated in an on-demand fashion by means of a special radio signal and a WuR receiver. In this paper, we analyze a novel wake-up radio approach that integrates both data communication and wake-up functionalities into one platform, providing a reconfigurable radio operation. Through physical experiments, we characterize the delay, current consumption and overall operational range performance of this approach under different transmit power levels. We also present an actual single-hop WuR application scenario, as well as demonstrate the first true multi-hop capabilities of a WuR platform and simulate its performance in a multi-hop scenario. Finally, by thorough qualitative comparisons to the most relevant WuR proposals in the literature, we state that the proposed WuR system stands out as a strong candidate for any application requiring energy-efficient wireless sensor node communications.

## 1. Introduction

As wireless networks are involved in our daily lives more and more, the challenges they bring get more pronounced. Clearly, an important challenge is the limited battery life of the wireless devices, *i.e.*, the longest time that a single battery charge may last. This challenge gets even more important for constrained devices such as wireless sensor nodes, which often feature minimum to zero user intervention.

Since wireless communication is a dominant energy consuming operation, achieving the most energy efficiency in this aspect has been a main target of the research community. A trivial approach for this is to reduce the energy waste caused by the radio communication, two important reasons of which are *idle listening* and *overhearing*. Idle listening occurs when a node is in reception state but no communication is present in the channel, while overhearing occurs when a communication is received by a node, yet it is not intended for that node. The conventional solution proposed and applied for reducing the idle listening has been *duty-cycling*, where the nodes are put to sleep regularly. In this approach, they are not in receiving state permanently, whereas they wake up regularly to check for potential incoming packets. However, although the duty-cycling mechanism reduces the idle-listening, it does not fully remove it, yet causing considerable energy waste and sleeping delay.

**Figure 1.**
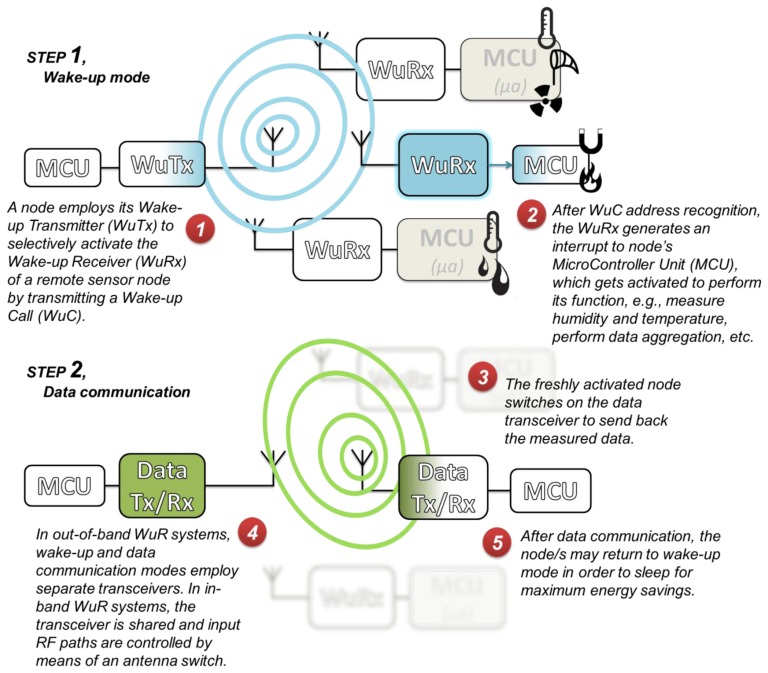
The Wake-up Radio paradigm.

Quantitative evaluations, e.g., [1], show that a more efficient alternative for wireless communications is the use of *Wake-up Radio* (*WuR*), which enables wireless devices to remain in very low-power deep-sleep mode, unless woken up by remote triggering. In WuR systems, as illustrated in Figure 1, a node initiating a communication activates the destination node in an on-demand manner by means of a *Wake-up Transmitter* (*WuTx*) sending a Radio Frequency (RF) signal, referred to as *Wake-up Call* (*WuC*), to the *Wake-up Receiver* (*WuRx*) attached to the intended node. Until the reception of the WuC, the node's MicroController Unit (MCU) and main data communication radio remain in deep sleep. This *wake-up* approach can be used in different applications, e.g., to retrieve information from environmental pollution sensors placed in a city by a mobile collector node, or to activate a sleeping wireless Access Point.

Wake-up Radio systems reduce or even eliminate the aforementioned energy inefficiency of duty-cycling. Numerically, this represents an important benefit since during the active period of duty-cycled nodes, where listening and transmitting take place, the node's MCU and main data transceiver present current consumption values in the order of mA [2,3]. Even for low duty-cycle settings such as 1%, this behavior implies constant energy waste during the on-state. In contrast, WuR systems allow nodes to present a constant current consumption value in the order of μA during the time their intervention is not required, significantly reducing the energy waste caused by idle listening. In addition, if a WuR system features an addressing mechanism also the overhearing issue is resolved, since it is possible to wake up a single node among several of them. Finally, the wake-up procedure can be performed in a short time, which reduces the latency which duty-cycled systems suffer from.

There are several types of WuR systems. The SubCarrier Modulation (SCM) WuR system, evaluated in this paper, is based on a reconfigurable way of operation where the node's radio can be used as both main data radio and WuR, *i.e.*, as an in-band WuR. This contrasts with most other WuR platforms requiring two separate transceivers and antennae for such functions, *i.e.*, out-of-band WuR. In SCM-WuR nodes, the radio settings are different for the two operation modes and can be switched by software. As shown in Figure 1, in its *wake-up mode*, SCM-WuR transmitter and receiver nodes respectively set the radio to transmit/detect the special wake-up RF signal, *i.e.*, the WuC. Upon reception of a WuC, WuRx triggers the MCU of the receiving node to switch from a very low-power sleeping state to an active state. Next, nodes set their radio transceivers to *data communication mode* to communicate in a traditional fashion. Thus, SCM WuR systems allow sensor nodes to remain in a very energy-efficient mode as long as their intervention is not required, which provides drastic energy savings.

In this paper, we provide a detailed characterization of the SCM-WuR system through physical experiments and measurements, evaluating it for different performance metrics and comparing it to other state-of-the-art WuR systems. Traditionally, the aim of the WuR studies in the literature has mainly been the improvement of the energy consumption performance at the WuRx side, while other performance metrics that are important for user applications are rarely considered as an objective. Such other metrics include the wake-up range, the wake-up probability, the current or energy consumptions of both WuRx and WuTx, the wake-up delay and how each of the previous factors affect the system's applicability to real scenarios. We believe a characterization of these performance metrics is crucial for the assessment of feasibility and utility of WuR systems. In addition, and for the sake of fairness, a comparison for these metrics is conducted for different WuR systems proposed in the literature, instead of just among ones with similar characteristics and/or performance. As a result, this paper also provides the most comprehensive WuR state-of-the-art to 2013. Finally, along the paper we also comment actual WuR application areas and several novel ideas under research, such as multi-hop WuR capabilities or nodes equipped with solar harvesting solutions requiring battery no power to operate.

The rest of the paper is structured as follows. We introduce and describe the SCM-WuR system in Section 2. Next, in Section 3, we analyze its performance in terms of several metrics. Two real application scenarios of the SCM-WuR system are depicted and also analyzed in Section 4. Finally, we compare the performance of the SCM-WuR to state-of-the-art of nowadays WuR systems in Section 5. Section 6 concludes the paper.

## 2. SubCarrier Modulation Wake-Up Radio Systems

In order to be adequate for different types of applications, a good WuR design must enable long operational ranges, require very low or no power, present a reproducible and low-cost hardware design and preferably operate at the Industrial, Scientific and Medical (ISM) frequency bands. SCM-WuR design satisfies all these requirements and presents itself as one of the best WuR candidates in the literature.

**Figure 2.**
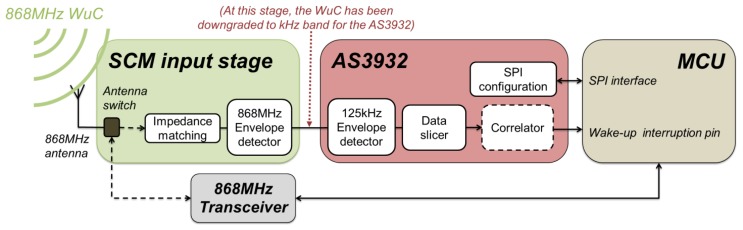
Block diagram of the SCM-WuRx/WuTx. Nodes route *Wake-up Calls* (*WuC*) and *data communications* by means of the antenna switch.

The SCM-WuR idea was first introduced in [4] and uses the off-the-shelf low-frequency (LF) AS3932 WuRx integrated circuit [5], which is a low-power Amplitude Shift Keying (ASK) receiver that is capable of generating a wake-up interrupt upon detection of a 110–150 kHz signal. SCM-WuR system design, illustrated in Figure 2, operates by switching between two radio modes that differ in their modulation schemes.

In *wake-up mode*, the WuRx detects the envelope of the 868 MHz WuC signal sent by a remote WuTx, which results in the underlying raw LF signal expected by the AS3932 chip. The AS3932 integrated circuit only requires a few μA of current to decode this LF signal. Afterwards, in *data communication mode*, an antenna switch is employed to bypass the AS3932 chip and communication is managed directly by the 868 MHz radio transceiver.

**Figure 3.**
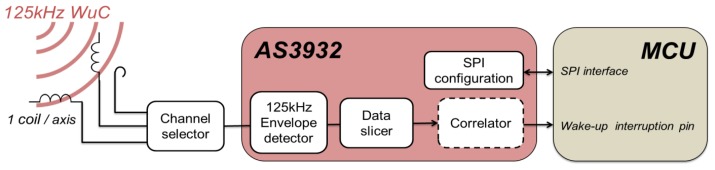
Block diagram of Austria MicroSystems WuRx. WuC is detected by magnetic coupling.

To incorporate the AS3932 into the SCM-WuR approach, it is important to clearly understand its working principles. Thus, they are described briefly as follows. In fact, the AS3932 chip itself may be used as a 125 kHz WuRx in short-range applications such as remote key locks, or automotive-related applications such as Tire Pressure Monitoring sensors. The block diagram of AS3932 WuRx board is shown in Figure 3, where the chip is connected to three input coil antennas that enable reception independent of the node's orientation. In such configuration, the AS3932 consumes 8.3 μA. In turn, for this AS3932's original system design, the WuTx requires up to +33 dBm (9V), because of WuR communication relying not on electric but on magnetic coupling. Unfortunately, such RFID-like approach results in limited operational distances of 5 m [6]. Upon reception of the amplitude-modulated WuC, a channel selector in the AS3932 routes the RF input of the coil antenna with the best reception to the 125 kHz envelope detector, which extracts the overall shape of the signal. Next, a data slicer operating at a specified bit-rate quantifies the amplitude values as “1” or “0”. Finally, a correlator checks if the bit pattern extracted from the WuC matches the particular address of this WuRx. Such address can be assigned to the AS3932 through its Serial Peripheral Interface (SPI). If the incoming address and the value in the internal correlator match, the chip generates an interrupt to switch the sensor node's MCU from sleep to active state.

The SCM-WuR design in Figure 2 reuses in an effective way the characteristics of the AS3932 integrated circuit, and deploys an 868 MHz +2 dBi gain omnidirectional antenna instead of the three coil antennas of Figure 3. By disabling two RF paths and the channel selector in Figure 3, the AS3932 current consumption is reduced from 8.3 μA down to 2.6 μA. After the antenna in Figure 2, an external envelope detector demodulates the 868 MHz WuC to extract the 125 kHz signal. Afterwards, the integrated 125 kHz envelope detector of the AS3932 extracts the original data from the 125 kHz carrier as in Figure 3.

**Figure 4.**
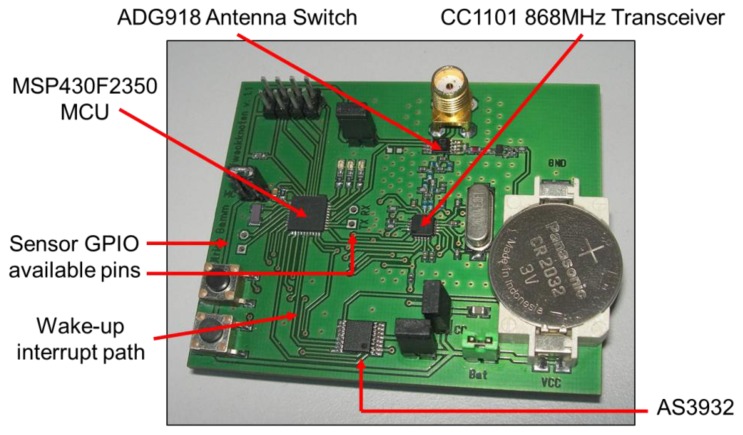
The SCM-WuR hardware board.

From the operational point of view, for a SCM-WuR node in *wake-up* mode, the RF switch, controlled by the MCU, is set to route the RF signal either from the 868 MHz transceiver to the antenna in the case of WuTx to generate a WuC, or from the antenna to the AS3932 in the case of WuRx. In traditional *data communication* mode, the antenna is directly connected to the 868 MHz transceiver. In our implementation, the Texas Instruments CC1101 868 MHz transceiver [7] is used. The hardware boards designed for the SCM-WuR system, shown in Figure 4, include a low-power MCU which enables the nodes to operate as either WuTx or WuRx. The node in the figure is powered by means of a simple coin cell battery with a capacity of 225 mAh (3 V).

**Figure 5.**
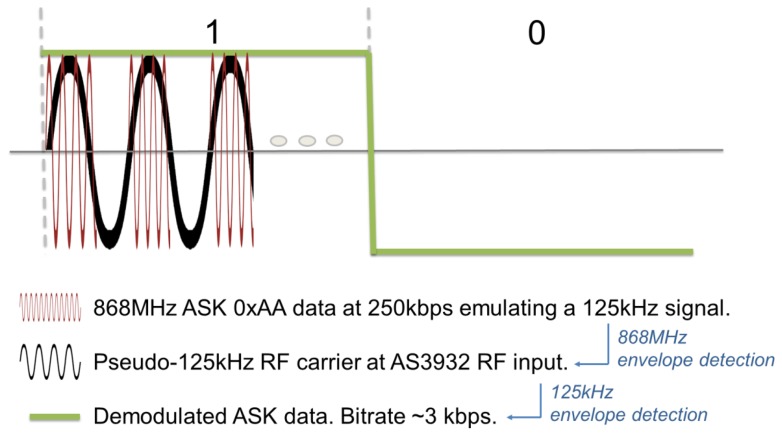
Bits of a WuC in a SCM-WuR system.

From the signal processing point of view, a double modulation strategy (also called SubCarrier Modulation or SCM) is used in *wake-up mode*; the WuC is modulated in OOK resembling a 125 kHz signal, where each cycle of this 125 kHz signal is modulated in OOK using an 868 MHz signal as a subcarrier. That is, to send each bit of information of the WuC, the MCU of the WuTx shapes a continuous RF wave at 868 MHz generated by the CC1101. To transmit a data bit value of “1”, the CC1101 transmits the bit sequence of 1010 … at 250 kbps, which appears to be 125 kHz signal after the envelope detector at the receiver, and each of these “1”s is shaped using a modulated 868 MHz carrier (Figure 5). To transmit a “0”, the CC1101 simply remains silent. To ensure the validity of such double modulation strategy, we program a signal generator to output an 125 kHz OOK modulation on a 868 MHz carrier, and connect it directly to the WuRx board using a SMA cable. Down to a signal ouput level of −53 dBm of the signal generator, the Signal to Noise Ratio (SNR) lies in a range where the WuRx reliably detects the WuC. The idea behind the use of such double modulation for the WuC is to benefit from the reduced antenna size and better gain of sub-GHz frequency signals, and overcome the short operational range problem of the WuRx design in Figure 3 which operates in the 125 kHz frequency band. When in *data communication mode*, the node transmits a typical wireless data frame containing measures from any sensors attached to any of the several General Purpose Input Output (GPIO) pins available.

In the SCM-WuR system developed, the last bits of the WuC contain the address of the intended receiver node. A WuRx generates an interrupt only if the address in the WuC matches the address set for that WuRx. WuRx can use unique address assignments or role-based assignments. For example, all router or relay devices can be assigned a specific role identifier. In a similar way, a WuC can be employed to activate only nodes attached to an intended sensor type, which can be humidity, fire, garbage, heart-rate, pressure, *etc.*

SCM-WuR can be implemented without an AS3932 integrated circuit, since the main component it employs is an address correlator. Other WuRx proposals in the literature implement similar correlators by means of shift registers and parallel comparators. However, since the AS3932 already contains an efficient address correlator as well as a kHz envelope detector, such integrated circuit is conveniently reused and deployed in SCM-WuR boards for a high-performance and reproducible design. Another important feature of SCM-WuR is that by deploying adequate envelope detection stages in the RF input path, SCM-WuR systems working at other frequencies, such as at 433 MHz or at 2.4 GHz, can easily be implemented, which makes the SCM-WuR design very flexible. Another advantage of the SCM-WuR is that it does not require two separate wireless transceivers for data communication and for WuR, reducing the overall monetary cost of the system.

## 3. Performance Analysis of the SCM WuR System

In this section, we present a complete performance analysis of the SCM-WuR system. The characterization is done for timing and wake-up delay analysis, current consumption of WuTx and WuRx boards at different communication stages, and operational distance ranges achieved for WuCs transmitted at different WuTx power levels.

### 3.1. Timing and Wake-Up Delay Analysis

**Figure 6.**
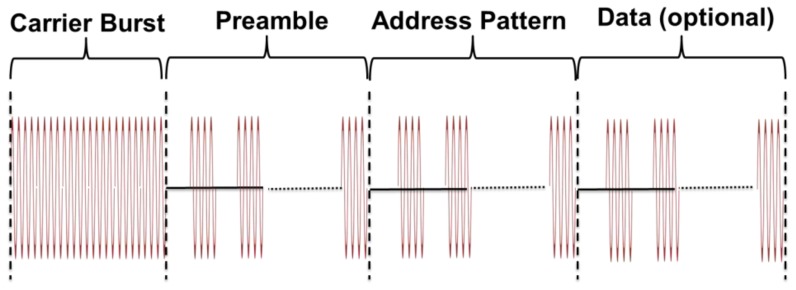
WuC format as in the AS3932 datasheet [5].

The AS3932 chip, found after the 868 MHz envelope detector employed for SCM-WuRx, expects the WuC to be sent according to the format in Figure 6. The WuC format consists of a carrier burst, a preamble containing several consecutive 0-1-0 bit transitions, an optional 16-bit address pattern and an optional data sequence. The data field allows two devices to exchange a small size data without leaving *wake-up mode*. If employed, such data reception requires the MCU to monitor one DATA pin in the AS3932. Low-power pin monitoring can be done by means of energy-efficient strategies like TicK [8], which requires a current consumption of few μA.

Addressing is also optional in SCM-WuR systems. If address pattern recognition is enabled, the AS3932 generates a wake-up interrupt on its WAKE pin for node's MCU if the entire WuC protocol is fulfilled and the address pattern matches the node's address. Instead, the AS3932 can also be configured as a plain frequency detector to further reduce energy consumption if addressing is not needed or, again, if alternative energy-efficient wake-up addressing strategies such as TicK [8] are considered. In such frequency-detection case, only the carrier burst is employed. In our evaluations, SCM-WuR boards are configured to use node addressing but no trailing data.

In the SCM-WuR system, the WuC bit-rate can vary from 1,024 bps to 8,192 bps. Such low bit-rate values are typical in WuR systems. We chose a bit-rate of 2,730 bps, since empirical evaluations show that sending WuCs at higher bit-rates results in a challenging signal for the envelope detector and data slicer of the WuRx, which leads to both operational range and WuC detection rate decrease. At such bit-rate of 2,730 bps, we measured the transmission of a WuC to last for 12.2 ms. In *data communication*, components are able to work at higher bit-rates than when used for WuR purposes. For example, the CC1101 is able to operate up to 600 kbps.

**Figure 7.**
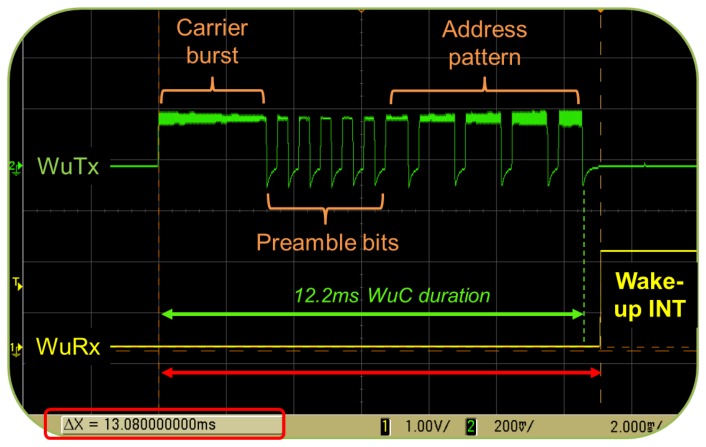
Incoming WuC from the SCM-WuTx and corresponding interrupt (INT) signal generated by the receiving SCM-WuRx to the node's MCU.

To quantify the total time required to activate an SCM-WuR board through a WuC, we measured the total wake-up delay by attaching one probe of an oscilloscope to the output of the WuTx and a second probe to the GPIO wake-up input of the WuRx's MCU. As shown in Figure 7, the wake-up delay is observed to be 13.08 ms, as a result of additional factors such as RF amplification settling time (250 μs) and the time for a bit to completely enter to the data slicer (366 μs for a bit-rate of 2,730 bps) to the WuC duration.

### 3.2. Current Consumption Analysis

The power level at which a WuC is transmitted presents a trade-off between energy consumption at WuTx and system's effective operational range. To quantify this trade-off, we evaluated three transmit power levels, −10 dBm, 0 dBm and +10 dBm, for which the corresponding current consumption values are measured to be 13 mA, 14.4 mA and 19.1 mA, respectively. Note that, as shown in Figures 6 and 7, half of the bits of a WuC are silent on average, thus the CC1101 requires less power to generate them. A WuTx is calculated to require 5.5 mJ to send a WuC.

Regarding the WuRx side, the CC1101 868 MHz transceiver is turned off in *wake-up mode*, while the antenna switch only requires a few nA, which is a negligible value compared to the current consumption levels in the μA order of the AS3932 chip (2.6 μA) and the MCU. The exact current consumption of the MCU depends on the Low-Power Lode (LPM) it is in. The MSP430F2350 MCU [9] employed in SCM-WuR boards can be configured to use LPM levels from 0 to 4. Different levels correspond to disabling/enabling the core, digital oscillators and different clock sources. Disabling all such MCU elements enable reducing the current consumption from 300 μA in active mode, and at an operating frequency of 1 MHz, to 0.1 μA when the MCU is in its deepest sleep mode LPM4, where it can only be activated by an external interrupt on one of its configured GPIO pins.

**Figure 8.**
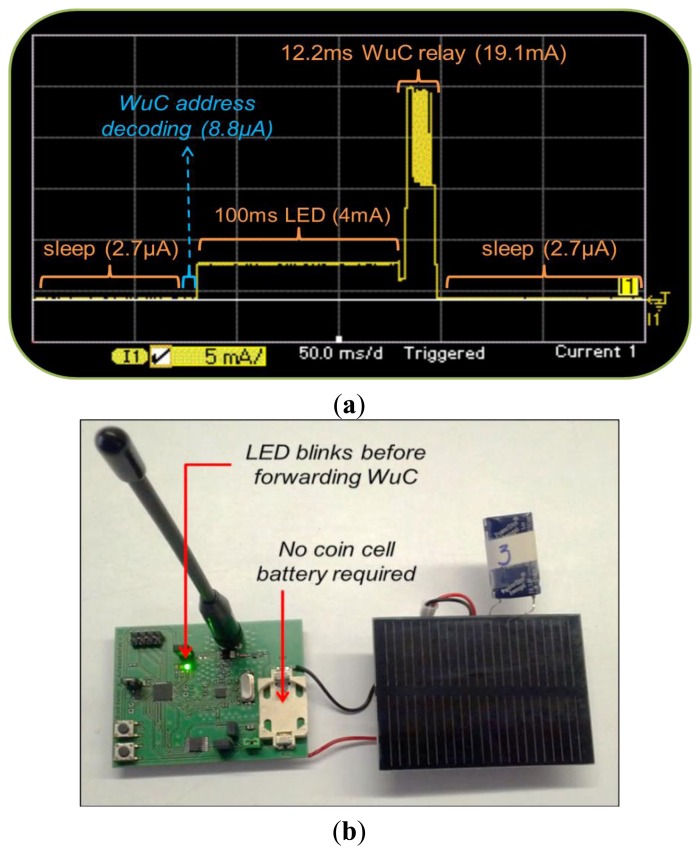
(**a**) Power analyzer trace of a SCM-WuR relay node; (**b**) Powering the relay SCM-WuR node by means of a solar cell and a supercapacitor.

To depict the stages of communication between WuTx and WuRx along the corresponding current consumption values experienced by SCM-WuR systems in detail, we employed an Agilent Technologies N6750A power analyzer. In the tests conducted, the SCM-WuR device is programmed to be initially in the WuRx mode. Upon reception of a WuC, it activates a LED for 100 ms and next switches from WuRx to WuTx role to send a WuC to a third node. As shown in Figure 8a, the WuRx in *wake-up mode* initially consumes as low as 2.7 μA (2.6 μA for the AS3932 + 0.1 μA for the MCU), since it is just waiting a possible incoming WuC carrier burst. When a carrier burst is detected the WuRx starts decoding the address, which requires 8.8 μA. Comparatively, under duty-cycling schemes (e.g., IEEE 802.15.4, IEEE 802.11 or 3G/4G) the mobile device is periodically activated to check for a possible incoming communication, requiring current consumption values in the order of mA, *i.e.*, 1,000 times higher than that of SCM-WuR. Such differences in current consumption become more pronounced for networks with large number of nodes, as the total energy savings get multiplied.

In Figure 8a, after the decoding of a matching address, an interrupt is sent to the MCU and a signaling LED is activated for 100 ms. Then, the CC1101 is activated (240 μs, not shown in the figure) and a new WuC is transmitted. As shown in Section 3.1, such WuC lasts 12.2 ms and requires 19.1 mA for an output power of +10 dBm. If the whole 16-bit addressable space is not required, the WuC duration value can be reduced by using node addresses ending with bit values of “0”.

The described application example represents a multi-hop wake-up through the use of SCM-WuR, which, to the best of the authors' knowledge, is being demonstrated in the literature for the first time in a real WuR hardware platform. A similar power profile is expectable for another type of application where a node sends back a transducer measure to the collector node originally sending the WuC.

The energy requirements of SCM-WuR nodes are so low that they can be powered by means of solar harvesting solutions. As a proof-of-concept, a novel functional prototype implementing the WuR multi-hop procedure of Figure 8a is shown in Figure 8b.

### 3.3. Wake-Up Range Analysis

A characterization of the operational distances achievable when employing different transmit power levels is essential to observe the trade-off between range and current consumption for the SCM-WuR system. This evaluation also enables devising adaptive transmitting power strategies which can achieve further energy efficiency. Unfortunately, most WuR proposals in the literature omit such range analysis.

In our test set-up of the wake-up range analysis, the WuTx is fixed at a coherent height of 1 m and the WuRx is displaced vertically and horizontally relative to the WuTx, in steps of 40 cm and 50 cm, respectively. For the WuTx, three transmit power levels are evaluated in this analysis: −10, 0 and +10 dBm. The SCM-WuRx is measured to feature a sensitivity of −53 dBm. In the evaluations, the WuRx is attached to a Bluetooth Low Energy (BLE) device. When the WuRx receives a WuC destined to it, it sends a wake-up interrupt to the input pin of the BLE device's 8051 MCU. BLE device's MCU is programmed to stay in low-power mode when idle, and to generate a single BLE *Advertising frame* every time it detects a wake-up interrupt on its input pin. These BLE reply frames are detected over the air by the use of a BLE sniffer. Thus, in this scenario the main data communication and the wake-up communication are done in different frequency bands, *i.e.*, it corresponds to an *out-of-band* WuR solution. If instead the data frame is transmitted back by means of an integrated transceiver, such as the CC1101 in case of the SCM-WuR boards, the WuR solution is considered *in-band*.

**Figure 9.**
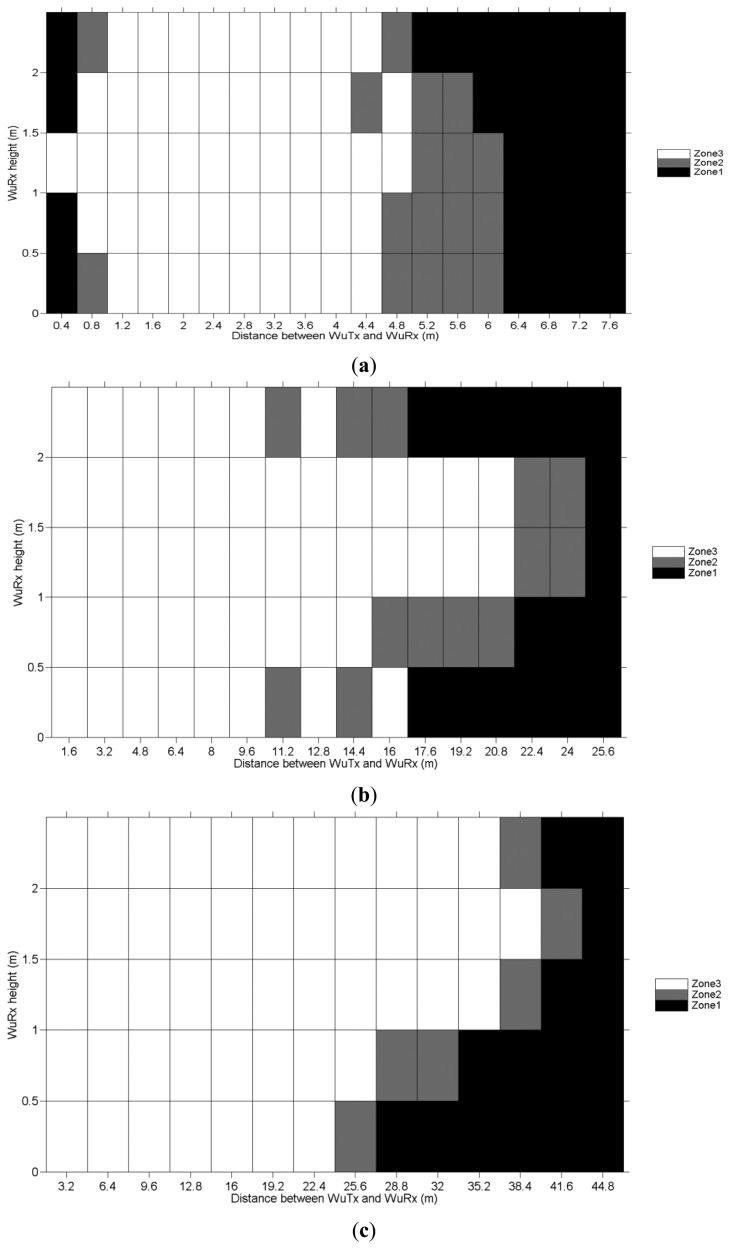
Wake-up distance evaluations for SCM-WuTx output power of (**a**) −10 dBm; (**b**) 0 dBm; and (**c**) +10 dBm.

The amount of sniffed BLE *Advertising Frames* matches the number of successfully decoded WuC. Based on this number, three operational zones are defined as shown in Figure 9: *Zone 1* denotes a consistent reception of the WuCs and is represented by white color; *Zone 2* denotes the zones with certain WuCs are detected, but reception is not 100% guaranteed and is represented by gray color; in *Zone 3* the WuRx is not activated at all by any WuC, which is represented by black color.

As shown in Figure 9, the maximum operational distance achieved is about 41 m for a WuTx transmit power of +10 dBm, which is a significant improvement over the maximum range of most state-of-the-art WuR solutions, as shown in Section 5. For the other two tested transmit power levels of −10 dBm and 0 dBm, the maximum operational distances measured are 6 and 24 m, respectively. A range of 6 m is adequate for several applications such as Wireless Body Area Networks (WBAN).

**Figure 10.**
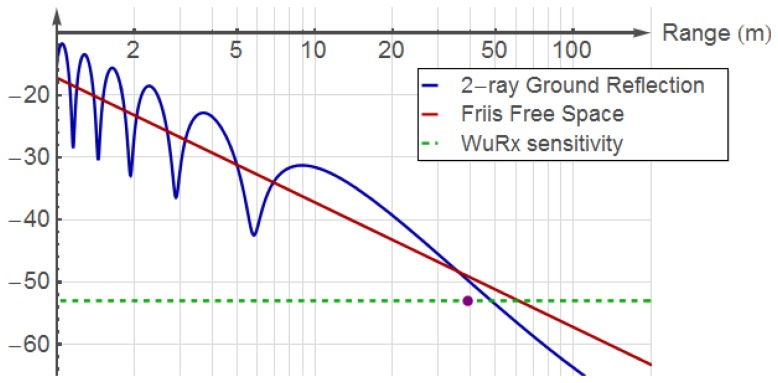
Simulation of theoretical received power of SCM-WuRx at different distances *vs*. its measured sensitivity.

In order to compare the theoretical range limit to the values featured by the SCM-WuRx, a Mathematica [10] simulation employing the values in Table 1 is conducted. The results in Figure 10 show both the theoretical free-space Friis distance and the 2-ray ground reflection model for the described scenario. The dot in the figure depicts the WuRx real operational range results from Figure 9c.

Since the maximum allowed transmit power at the 868 MHz band is +27 dBm in Europe, the wake-up range of SCM-WuR can still be increased by using amplifiers such as the CC1190, with the counterpart of increasing energy consumption at the transmitter side. For a +20 dBm output power, more than 100 m range has been measured for SCM-WuR systems. This operational wake-up range is among the longest ones in the literature for a WuRx current consumption of few μA [11].

## 4. Illustrative Application Scenarios and Multi-Hop Network Performance Evaluation of SCM-WuR

The characteristics of SCM-WuR make it suitable for a wide range of applications. In this section, we illustrate both types of scenario, one single-hop and one multi-hop, and evaluate the network performance of the latter by means of simulation.

**Figure 11.**
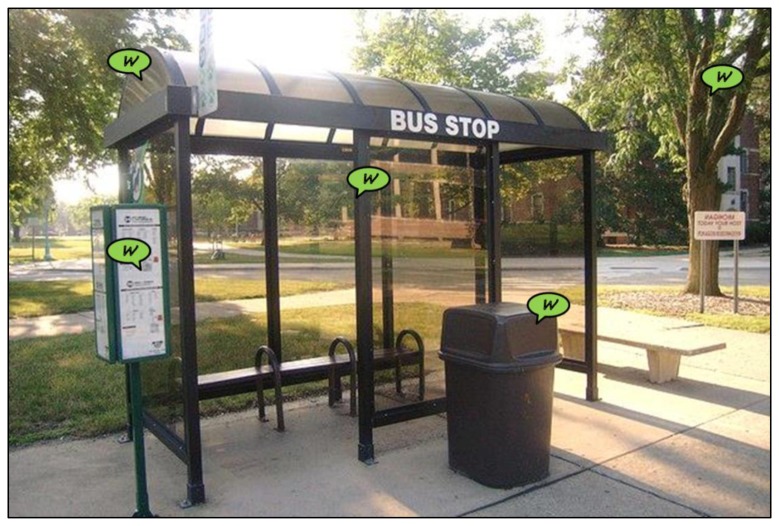
A bus stop equipped with SCM-WuR sensors.

A possible single-hop scenario is urban sensing, for which a *data-mule* application is considered. Under this scenario, a SCM-WuR board acting as a collector node is placed in a non-power-restrained mobile node, which can be a public bus or a garbage truck. Such mobile node travels around the city and queries SCM-WuRx-equipped sensors placed along its route. Thus, the only moment that the deployed SCM-WuR boards get activated is when the mobile node gets close. Such behavior differs from typical wireless sensors that perform duty-cycling the entire time, only to be queried when the data-mule passes nearby, which can correspond to once in several hours. In our scenario, remote sensors are placed or attached to any element in town, as illustrated in Figure 11. As shown in Figure 8b, in the case that such SCM-WuR sensor nodes are queried infrequently they can be powered by means of energy harvesting solutions, because the supercapacitor charges up between queries.

The trash bin in Figure 11 can host an SCM-WuR-equipped infrared sensor to transmit back the height of the garbage once queried. Also, trees can be equipped with humidity sensors in order to check they are being irrigated enough during the day. Even the bus stop can be equipped with environmental sensors such as solar radiation and pollution sensors. Finally, panels can be equipped with proximity sensors to be aware of how many users read the information, or advertisements, displayed in them. The SCM-WuR system can query any or all of these sensors by means of the proper addressing. Such addressing may be not unique, thus the same address can be set for all the sensors of the same type, e.g., humidity, in a role-based addressing fashion. Thus, the same address can be reused between several bus stops to just address certain types of sensors. A GPS-based application connected to the city database makes the mobile node aware of which kind of sensor is deployed in each area of the town. For this single-hop application, SCM-WuR boards capable of operating up to 40 m are adequate. As commented, this range can be adjusted and even reduced if shorter ranges are enough.

For the multi-hop scenario, a slightly different version of the SCM-WuR boards is considered to enable them operating up to distances of 100 m and/or traverse walls. For this, the boards are equipped with a CC1190 output power amplifier in order to be able to transmit up to +20 dBm. In this configuration, the current consumption featured by SCM-WuRx only slightly increases up to 3.5 μA, while the current consumption for a WuC transmission noticeably raises up to 152 mA. Currently, there are ongoing research efforts to reduce such high value for WuC transmission. The current consumption increase for boards in *wake-up mode* is due to a design modification to employ a CC430, which merges in a single integrated circuit both MSP430 and CC1101 components. While this change allows for less circuitry, the LPM4 for the CC430 is not as efficient as the one for spare MSP430 MCUs. This 100-m version of the SCM-WuR boards is powered by 2 AA batteries for a capacity of 1,500 mAh (3 V).

**Figure 12.**
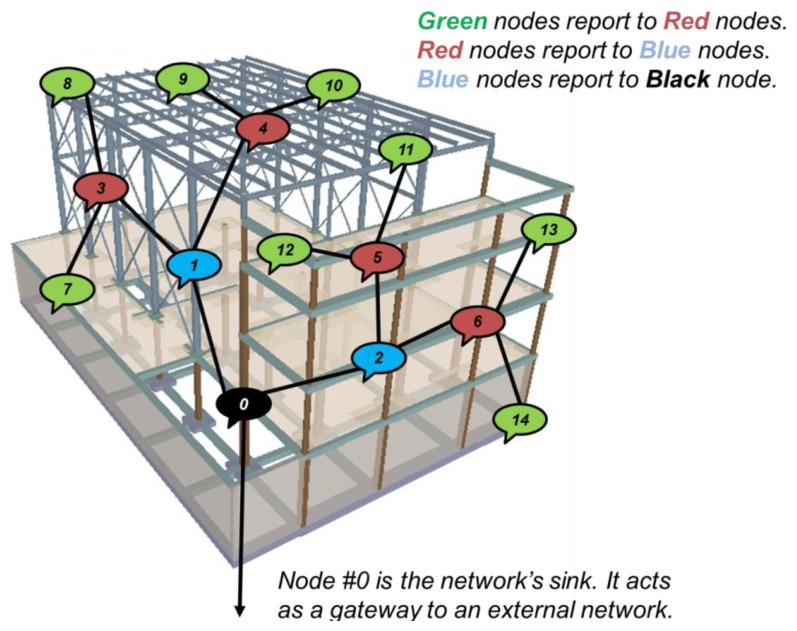
A network of SCM-WuR vibration sensor nodes in a multi-hop scenario.

In order to test the performance of the SCM-WuR approach under the multi-hop scenario, we developed an OMNET++ [12] model for our SCM-WuR boards and defined a convergecast tree topology depicted in Figure 12, *i.e.*, where several sensor nodes periodically send their vibration measurements to a sink. Intermediate nodes have to send their own measurements, as well as to relay the information they receive. In this scenario, the network's sink, *i.e.*, node #0, merely operates as a receiver. When an operator comes nearby, the sink provides all the information collected, possibly in an aggregated fashion.

**Figure 13.**
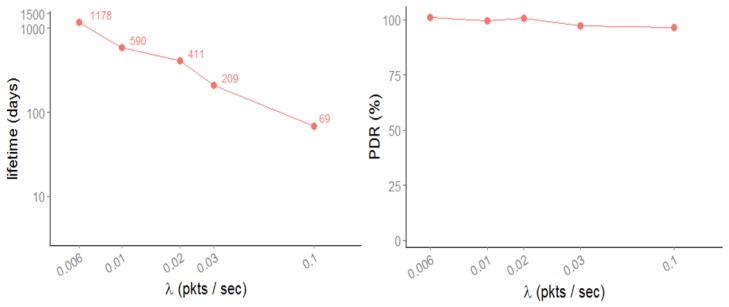
Lifetime in days (**left**) and PDR (**right**) of node #1.

SCM-WuR transmissions are able to traverse up to three walls along their RF path. Therefore, sensors can be placed anywhere in three dimensions along the scenario in Figure 12. In the simulation results of Figure 13, the *x*-axis represents packet rates (λ) of 0.1, 0.03, 0.02, 0.01 and 0.006 packets per second, which correspond to periods from 10 s to 180 s between packets. The simulation lasts for 10,000 s and up to 10 tests are performed for each packet rate. The scenario parameter settings used in the simulations are shown in Table 2.

Figure 13(left) depicts lifetime values achieved by one of the bottleneck nodes, node #1. Node #1, besides sending its own measures, is in charge of forwarding packets from half of the nodes in the network and hence, it is of the two most power-demanding nodes. As seen in the figure, node #1 lifetime values decrease as more frequent sensor reports are used. For periods longer than one minute (λ∼0.016), SCM-WuR network in Figure 12 allows for lifetimes longer than two years, although in a real application this value could vary depending on the quality of the batteries when providing current peaks. Each report from a sensor in the network consists of one WuC followed by one regular data transmission, which approximately requires 12 ms (WuC) + 3 ms (data) = 15 ms channel access time. Numerically, for a reporting period of 180 s, a SCM-WuR node is active during 0.000083% of the time. In contrast, duty-cycled systems may be active up to 5% of the time [3]. When the reporting period decreases, WuR lifetime performance gets closer to the ones typical for duty-cycled solutions (e.g., lifetime is approximately 69 days for a reporting period of 10 s or λ = 0.1 packets per second). However, for many real-life applications reporting periods of at least several minutes are expectable.

The successful Packet Delivery Ratio (PDR) of the sensor readings to the sink is depicted in Figure 13(right). As shown, for reporting periods longer than 10 s (λ = 0.1 packets per second) the PDR for SCM-WuR in this scenario is practically 100%, *i.e.*, all packets sent by all nodes are successfully transmitted to the sink. A shorter reporting period results in a decrease of the PDR, which shows the drawback of WuR systems for applications that require very frequent node wake-ups.

## 5. SCM *vs.* State-of-the-Art WuR Approaches

In this section, a comparison between the most relevant state-of-the-art WuR proposals and the SCM-WuR approach is performed in terms of current consumption, circuit complexity, operational range, addressing, application flexibility and WuC bit-rate. To convey the overall picture of the state-of-the-art and to position the SCM-WuRx approach accordingly, we categorize the WuRx proposals based on their distinctive features. Information for respective WuTx is provided when available. Along the section, we employ both *active* and *passive* terms to indicate if a WuRx requires a battery to operate or not, respectively. At the end of the section, a global comparison table summarizes the most important characteristics of each approach (Table 3).

### 5.1. Radio Frequency IDentification (RFID)-Based WuR Proposals

**Figure 14.**
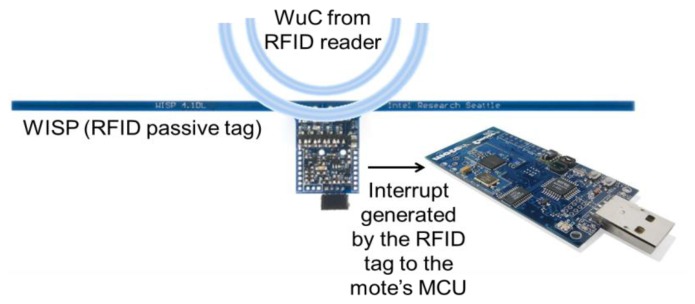
Passive RFID-based WuR system.

RFID technologies can be used as a basis for WuR systems, where the WuRx is an RFID tag and the WuTx is an RFID reader. However, their applicability and performance are strongly interrelated with the intended application. A passive RFID-based solution is presented in [1], where a programmable RFID tag, so-called Wireless Identification Sensing Platform (WISP), is powered by an off-the-shelf UHF RFID reader to generate an interrupt on an input pin of a Tmote Sky wireless sensor mote [13]. As shown in Figure 14, the WuRx generates an interrupt on a GPIO pin of the mote's MCU it is connected to upon detection of a WuC. When the MCU gets activated, it transmits an IEEE 802.15.4 wireless frame back containing the sensor data by means of the mote's CC2420 2.4 GHz transceiver.

Since the power required by the WISP's MCU to operate is provided by the magnetic field from the RFID reader when generating the WuC, the passive RFID-based WuRx proposal in [1] presents unbeatable power consumption values. Unfortunately, RFID-based WuR systems cannot provide operational distances larger than the typical ones for UHF RFID systems, about 5 m [1], an issue which limits their applicability. In addition, RFID readers, *i.e.*, the WuTx, require power in the order of watts to achieve such distances, and are considerable in size.

Other active RFID-based WuR proposals, such as RFIDImpulse [14], feature better WuTx current consumption and operational distance values of up to 30 m, but employ commercial *active* RFID tags as WuRx. Such tags require higher current consumption than other state-of-the-art WuRx approaches and cost as much as an entire wireless sensor mote, which represents a downside for WuRx designs [15]. Besides, the proposal in [14] strongly relies on the capability of a 2.4 GHz TagSense mote to perform WuC detection by means of a low-power Clear Channel Assessment (CCA). However, the applicability and performance of such mechanism are called into question in other studies [16]. In addition, it is not clearly stated if the proposal in [14] is a theoretical analysis, a simulation or a real implementation of a WuR system.

Medium-range applications appear to be the most suitable for RFID-based WuR systems. Such medium-range applications are related to *data-mule* scenarios, where a non-power-restricted mobile node, equipped with a RFID reader acting as the system's WuTx, travels around the application area to retrieve information from small sensors equipped with RFID-WuRx. This is the case of warehouse inventory or medical applications. One example of the latter case is that, instead of using traditional wired health monitoring devices, nursing staff of a hospital can easily gather all the sensor data from tags attached to patients' sensors by just entering their room and activating the RFID-WuTx. This requires a range of few meters, which RFID-based WuRx are capable to achieve [17]. It is also important to note that RFID-WuR systems rely on magnetic rather than electric coupling for communication. Therefore, certain application areas may be restricted to RFID-based WuR because of security or health concerns.

Thus, even if they feature the lowest current consumption, and mostly because operational ranges related to RFID are closely related to antennae sizes, RFID-based WuR systems suffer from having a restricted number of application areas. Because of this, small RFID readers are typically employed in short-range applications like access control systems, where the user approaches the tag for the communication to take place. Moreover, the use of RFID-WuR systems in small size communication devices is not possible because of the RFID-WuTx power requirements and antenna size. For example, an Ultra High Frequency (UHF) antenna is around 25 cm^2^ in size and the associated reader may require up to +30 dBm [18] to activate a remote RFID tag from several meters away.

Comparing SCM-WuR with RFID-WuR, the advantage of RFID-WuR is the no-power consumption at the receiver side for the passive RFID-tag based solutions. However, as seen in the literature, the achievable operational distance of this method is very limited. On the other hand, SCM-WuRx featuring about 3 μA and long operational distances seem to enable many more use cases. Furthermore, such small absolute current consumption value can achieve very long lifetime for low data-rate applications, as described in Section 4. Also, as shown in Figure 8b, SCM-WuR nodes can even be powered by means of energy harvesting, which reduces the requirement of SCM-WuR to zero-power. Regarding operational distances, a SCM-WuTx only employs 0 dBm (1 mW) to wake-up a SCM-WuRx at 10 m away (Section 3.3), thus medical applications as in the RFID-based WuR system are perfectly feasible with a much lower power budget at the transmitter side than in the RFID case. Finally, SCM-WuR nodes also feature much smaller form factor for WuTx and can perform peer-to-peer communications, which are crucial requirements for other applications.

### 5.2. Heterodyne WuRx Proposals

**Figure 15.**
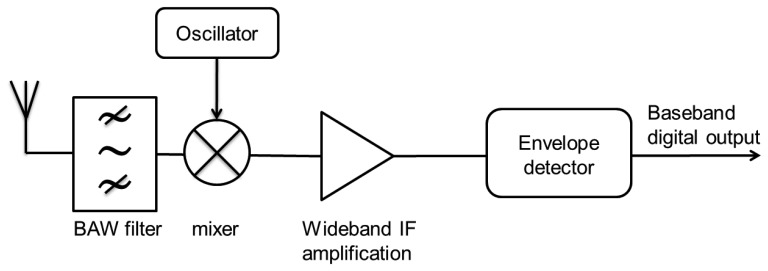
An example of heterodyne WuRx [19].

Heterodyning is the process by means of which an incoming radio frequency, a WuC in case of WuR systems, is reduced by means of signal mixing to an intermediate lower frequency that is easier to be processed by electronic components. A 2 GHz heterodyne proposal with uncertain (1 MHz to 100 MHz) IF (Intermediate Frequency) capable to operate up to bit-rates of 200 kbps is analyzed in [19]. In the proposed WuRx (Figure 15), the WuC passes through a Bulk Acoustic Resonator (BAW) filter, then its frequency is down-converted to IF by means of mixers feed by a ring oscillator. Next, the signal is amplified and filtered again. Finally, the WuC envelope is extracted and presented as a baseband digital output in one of the inputs of the node's MCU. The heterodyne design in [19], while achieving good sensitivity values of −72 dBm, implies a WuRx's current consumption value of 104 μA. Such value, even in the sub-mA order, is much higher than other state-of-the-art approaches.

A different heterodyne proposal for a crystal-less 2.4 GHz WuRx is presented in [20]. The WuC is modulated by means of Pulse Position Modulation (PPM). In order to reduce the power consumption of this WuRx, both the signal front-end and the oscillator are duty-cycled at the pulse level. However, the design still requires up to 350 μA.

Another heterodyne WuRx proposal that operates at the frequency of 45 MHz is presented in [21]. The performance of this design is evaluated by simulations. The WuRx targets WBAN applications, thus its purpose is to collect biometrical measurements after being activated. For signal amplification, the design employs an energy-efficient Injection-Locking Ring Oscillator (ILRO) instead of high gain RF amplifiers. At the final stage of the front-end, the signal is processed by a low power Phase Locked Loop (PLL) demodulator. The WuRx in [21] features a sensitivity of −62.7 dBm and, similarly to [19], it can operate up to 200 kbps, but still cannot reduce its current consumption to less than 53 μA.

As shown in the previous heterodyne approaches, it is common and straightforward to try to include few active components in WuRx designs in order to enhance them with better sensitivity or operational ranges than those of passive WuRx. However, WuRx containing active components tend to require excessive amount of energy. In fact, heterodyne approaches feature the highest power consumption and circuitry complexity among all the WuR approaches because of their active components such as mixers and amplifiers. Such design paradigm, while enabling good sensitivity and bit-rate values, implies current consumption values of up to 50 μA [21], 104 μA [19], and 350 μA [20], which are out of the desired range for competitive WuRx designs. Nowadays, WuRx are commonly required to operate under the 10 μA threshold. Therefore, it seems that heterodyne approaches are not enough energy-efficient for WuRx designs. Furthermore, most heterodyne WuR proposals operate at non-ISM bands such as at 2 GHz [19] or at 45 MHz [21], a circumstance which potentially would require the WuR system to work out-of-band. This, in turn, often implies that the wireless sensor requires a second transceiver operating in ISM frequency bands.

Comparing heterodyne approaches with SCM-WuR is challenging, since most of the studies lack information about their application areas, addressing capabilities and/or achievable operational distances. For example, heterodyne WuRx proposals in the literature output a baseband sequence when receiving a WuC, thus they are not considered to feature embedded addressing. Despite this, they can still be compared for other metrics, such as bit-rate. In fact, heterodyne WuR system proposals provide WuC bit-rates noticeably higher than SCM-WuR. Nevertheless, because of the main function of a WuRx consists of activating an intended sleeping sensor node, SCM-WuR systems do not feature the hundreds of kbps of heterodyne WuR systems, thus can discount the active components. Such circumstance allows SCM-WuRx designs for less complicated and cheaper circuitry, which results in designs requiring up to ten times less power. Also, SCM-WuR systems operate *in-band* at 868 MHz, thus there is no need for additional data communication transceivers.

### 5.3. MCU-Based WuRx Proposals

**Figure 16.**
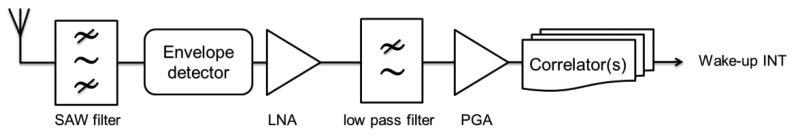
Employing a MCU in a WuRx design.

These proposals employ an additional independent MCU in a WuRx to perform several tasks such as signal filtering. Unfortunately, such approach is both agile to implement and inefficient in terms of energy consumption. As an example, Figure 16 depicts an AT-mega128L MCU, which decodes a WuC after signal rectification and amplification. The AT-mega128L is always kept in active mode requiring 801 μW at 3 V [22]. If this MCU decodes the proper node address in the WuC, it wakes up a second and more powerful MCU from its sleep mode. Before the AT-mega128L, the design in [22] features an energy-hungry but not very efficient amplifier stage, which provides operational ranges of barely 3 m.

Because of requiring an always-active MCU, its high current consumption value and the mentioned limited operational range, the design in [22] is not suitable for WuRx purposes. SCM-WuR clearly outperforms WuR systems based on a secondary MCU [22] in every metric. Clearly, MCU-based WuR systems cannot be considered for realistic applications, yet they are presented in this paper as an alternative approach existing in the literature.

### 5.4. Low-Complexity WuRx Proposals

The performance achieved by both previous approaches, heterodyne and MCU-based, shows that precisely the most effective way to reduce energy need is trying to simplify the circuitry in a WuRx.

Chronologically, the first proof-of-concept WuRx is presented in [23], simply featuring a capacitor and a rectifying diode. Interestingly, this simplistic and low-complexity WuR system even considers an addressing scheme, where WuTx transmits signals at different frequencies simultaneously to activate an intended WuRx. However, this WuR system proposal features poor operational ranges of few meters.

Another low-complexity 868 MHz WuRx consisting of a voltage multiplier, a rectifier scheme and a voltage comparator is presented in [24]. The WuR system, while featuring an interesting WuRx current consumption value of just 900 nA, does not achieve operational distances larger than 3.5 m and is prone to false wake-ups caused by RF interferences at the mentioned frequency. To overcome these limitations, the authors in [6] propose several system improvements, such as adding a Surface Acoustic Wavelength (SAW) filter to protect the WuRx from being activated by interferences or different WuTx designs to increase the operational distances up to 15 m, a value that effectively allows the WuR system to be employed for medium-range applications.

Another low-complexity WuRx proposal in [25] can selectively operate at 915 MHz or 2.4 GHz depending on the chosen input stage configuration, which can be varied by means of different input coil configurations at the RF impedance matching stage. The described WuRx consumes 51 μA at 1 V and basically amplifies the WuC signal, extracts its envelope and if a duty-cycled Analog to Digital Converter (ADC) considers the signal powerful enough, generates an interrupt destined to the node's MCU. Similarly, the WuRx presented in [26] splits its operation in a two-step fashion, the former one of which is also duty-cycled. In *monitoring mode*, WuC is detected by means of a duty-cycled comparator. This mode consumes as low as 4.7 μA from a 1.8 V power supply. Afterwards, upon detection of a WuC, the WuRx is switched to *identification mode* to decode the address in the WuC. Unfortunately, the high bit-rates employed by this WuR system drive this address-decoding procedure to require up to 599 μA.

**Figure 17.**
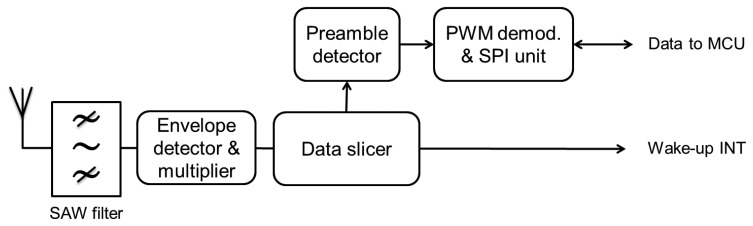
The low-complexity WuRx in [27].

The most efficient low-complexity WuRx proposal to date is presented in [27]. The WuRx, which block diagram is shown in Figure 17, is designed for WBAN applications. The design provides operational ranges of up to 10 m for a WuTx output power of +10 dBm. Indeed, the WuRx in [27] features one of the lowest current consumption of the all WuRx approaches (180 nA at 1.5 V), while still presenting a good trade-off between current consumption, hardware complexity and operational range. However, the WuC addressing feature is left to the MCU and its related current consumption is not mentioned in the paper. In fact, the WuC is used to trigger the data slicer and the Pulse Width Modulation (PWM) demodulator which provide a SPI translation of the incoming data to the MCU.

Yet another low-complexity WuRx design for WBAN is presented in [28]. Even though it also presents a minimal current consumption value of 82 nA at 1.2 V, the proposed 915 MHz design barely achieves operational distances of 1 m for 0 dBm. Thus, this latter WuRx is not considered for the comparison in this paper.

**Figure 18.**
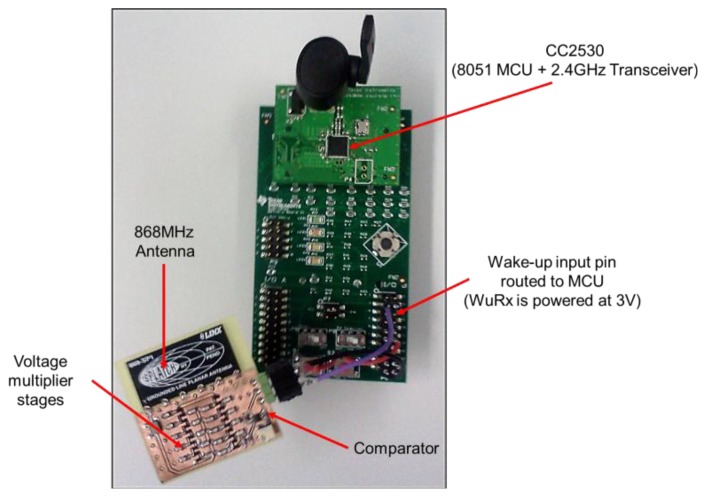
The low-complexity WuRx in [6] attached to a wireless sensor node. When the WuRx detects a WuC, the main board is woken up from sleep mode to transmit back an IEEE 802.15.4 data frame.

Low-complexity WuR designs can be considered as an intermediate approach that combines the best of RFID-based and heterodyne approaches, since they feature less circuitry complexity than heterodyne approaches and, at the same time, better ratio between operational range and current consumption than RFID-based WuR systems. In addition, most low-complexity WuRx proposals provide full details in order to make designs reproducible. Low-complexity WuR systems also operate at bit-rates much more adequate for WuR purposes than those of heterodyne approaches. For example, the low-complexity WuR system in [24] fits in the short-range application area, like RFID-based WuRx designs. However, since the related WuTx can be implemented by means of a RF transceiver instead of a RFID reader, it presents much better possibilities in terms of peer-to-peer applications,. In addition, in case more efficient antennae are allowed in WuTx [6] as in the RFID case, the WuR system may feature higher operational range values than that of RFID-based WuR. Such potential for larger operational distance values, along with a current consumption value as low as 0.9 μA, enables the WuRx to be employed in a larger number of applications. Figure 18 shows a wireless sensor platform based on the Texas Instruments CC2530EM [29] board equipped with a custom implementation of the low-complexity WuRx described in [6]. The design is empirically checked to feature operational distances around 10 meters by the same procedure in Section 3.3.

The low-complexity WuRx proposals in [25,26] duty-cycle several WuRx components to reduce the current consumption. Such strategy cannot be performed without affecting WuTx transmissions, which needs longer or repeated transmissions to ensure the duty-cycled WuRx is able to detect them, as in B-MAC [2]. Clearly, both circumstances imply either higher current consumption at the WuTx side or a reduction in the performance in terms of packet delivery, which may be not acceptable in certain WuR applications. Another drawback among all low-complexity WuR proposals is that they either lack addressing capabilities or require high amount of current to perform the address decoding procedure (up to 0.6 mA in [25]).

Unfortunately, low-complexity WuR proposals [25–27] tend to lack range-related information, thus their comparison to SCM-WuR is difficult. However, since their design includes few active components, operational distances around 10 m would be expectable. For example, the WBAN low-complexity proposal in [27] is stated to achieve 10 m for a WuTx output power of +10 dBm. However, due to the fact the WuTx already employs the maximum allowed power at 433 MHz, the design cannot achieve longer operational ranges. Compared to [27], SCM-WuR provides noticeably higher operational ranges and slightly higher sensitivity (−51 dBm *vs*. −53 dBm). As shown in Figure 9c, the maximum operational distance achieved by SCM-WuR is about 41 m for a WuTx transmit power of +10 dBm.

While low-complexity WuR systems present an evolution when compared to RFID-based, heterodyne and MCU-based ones, their limited operational distances and the fact that they lack an address correlator result in important performance issues. Instead, SCM-WuR systems deploy a correlator expressly devoted to decode address embedded in WuC while still featuring very few μA. In addition, the SCM approach allows for real operational ranges up to 100 m. Finally, SCM-WuR boards do not present duty-cycled components in their design. These characteristics allow SCM-WuR systems to be more efficient and adequate for a wider range of application scenarios than low-complexity WuR.

### 5.5. Correlator-Based WuRx Proposals

**Figure 19.**
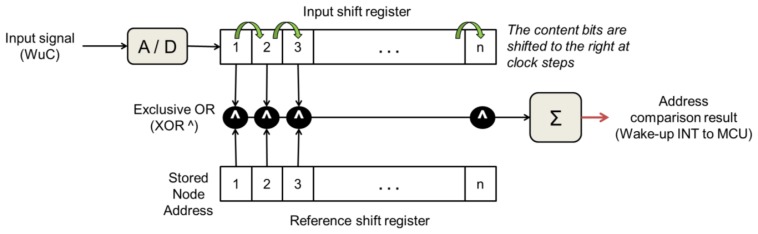
Address comparison by means of bit correlation.

The most efficient WuRx designs in the literature employ hardware correlators. Internally, a correlator circuit generates a parallel output of the bits contained in a buffer, which are shifted at each clock transition with a new bit incoming through the signal input. Such output value is compared to a pre-stored one. The input shift register progressively hosts the demodulated address extracted from a WuC by analog to digital conversion (performed by envelope detection in most WuRx) and compares it with the node's own identifier. If at any time both values are the same, the wake-up interrupt output pin is asserted. Figure 19 depicts the use of a correlator to decode an address in a WuC.

Address correlators circuit consume few μA when in idle state and about 10 μA when fully active decoding the WuC address [5,27]. Thus, all correlator-based WuRx designs implicitly feature addressing capabilities. For example, SCM-WuR boards require 8.8μA for their correlator to decode a WuC address.

A simulated correlator-based WuRx approach can be found in [30]. It features a current consumption value of 19 μA and operational distances of 4 m for a 0 dBm output power. This work is the basis of several newer proposals, since it depicts a complete WuC signal processing trace including all the sequential steps from envelope detection to the address comparison stages. Another simulated WuRx, but based on a Field Programmable Gate Array (FPGA), is presented in [31]. Because of both works being proposed only by simulation and featuring similar characteristics, we only consider the proposal in [30] for the comparison in this paper.

A real correlator-based WuRx employing a FPGA for decoding the WuC is presented in [32]. It requires 8.4 μA and is powered by 1.5 V power supply. Prior the FPGA, the design deploys an envelope detector and a programmable amplifier. Unfortunately, the operational ranges achieved when varying the amplifier gain are not provided in the paper. Since the WuRx is stated to target short-range applications, probably distances from 3 to 10 m are expectable.

**Figure 20.**
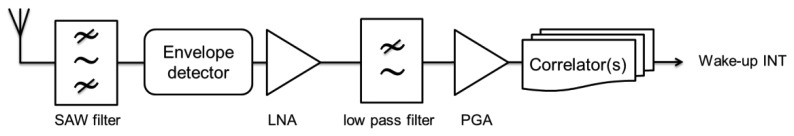
Block diagram of the correlator-based WuRx in [33].

The most efficient WuRx as of 2013 is presented in [33]. Such 868 MHz WuRx is based on a correlator capable of identifying addresses up to 64 bits in length. The power supply is 1.0 V and the total power consumption it features is 2.4 μW. The study in [33] includes the total time for the WuRx to fully activate the MCU, which takes from 40 to 110 ms. This WuRx is stated to feature operational distances of up to 304 m for a WuTx transmit power of +6.4 dBm. However, the proposed design, shown in Figure 20, lacks of crucial details of the WuRx, and is presented as a *black box*. Because of this, the proposal in [33] is hardly reproducible and analyzable.

Because of the proposal in [30] merely simulates a WuRx circuit, its performance cannot be compared to SCM-WuR directly. Hence, the most relevant WuR proposals in the category of correlator-based WuRx to be compared to SCM-WuR are [32,33]. The authors in [32] state the WuC address decoding is performed in FPGA for the sake of flexibility. This means the 8.4 μA consumption value featured by the WuRx can be reduced if addressing is implemented on-chip. Precisely, such modification is already deployed in SCM-WuR boards, which only present a value of about 10 μA when decoding of a WuC address. Instead, during idle state, SCM-WuR boards feature a third of the current consumption of the proposal in [32]. Despite this, both designs can be considered similar, even if the proposal in [32] is designed with short-range applications in mind. In fact, because of decoding of the WuC being performed by FPGA, the proposal in [32] even outperforms SCM-WuRx in few metrics such as WuC bit-rate capabilities of up to 100 kbps, which in turn means shorter WuC latencies. As a drawback, a WuRx design featuring a FPGA may present higher monetary cost. When compared to other high bit-rate WuR systems such as heterodyne ones, the FPGA-based proposal in [32] enables a drastic improvement in terms of current consumption. However, usually such high bit-rates are neither common, nor required for WuR applications.

On the other hand, the high performance WuRx presented in [33] features an 868 MHz impedance matching stage based on high-quality inductors for increased sensitivity as in SCM-WuR boards, along with operational ranges of 300 m for WuTx transmit power values below +10 dBm. Unfortunately, the design features a hardly reproducible circuitry due to the lack of details in the paper. In addition, the transmission of the WuC in [33] lasts for 4 to 10 times longer than that of SCM-WuR. Since during the WuC decoding WuRx cannot handle any other input RF signal, such WuC time duration may seriously affect the PDR of a network comprising several WuRx-equipped nodes.

### 5.6. Other Types of WuRx

Apart from the radio frequency WuR systems, there are also proposals based on different transmission medium. For example, the proposal in [34] utilizes Free Space Optical (FSO) for WuC communication. It achieves operational ranges of up to 20 m for a WuTx power of 16.5 mW. The wake-up receiver requires up to 100 μA. Unfortunately, the system suffers from low bit-rates of 2 kbps even in *data communication mode* and requires Line-of-Sight (LoS) between nodes. In [34], the authors state that, in the case of optical wake-up systems, addressing is inherently implemented by means of the directional nature of the optical medium. Yet, it is not clear the performance of this system for networks where nodes are not perfectly aligned.

Another FSO proposal is presented in [35], which features an ultra-low power consumption of 695 pW. This wake-up receiver allows for operating distances of up to 50 m when employing a fixed-position 3 mW laser as WuTx, of 6 m when employing a 3 W focusable LED and of 20 cm when employing a 0.5 W standard LED. Similarly to the design in [34], the wake-up system in [35] suffers from LoS requirements, even lower bit-rate (91 bps) and WuC detection capabilities extremely dependent on the physical alignment between the optical transmitter and receiver nodes. Each of these issues limits the application areas of optical systems. However, and differently from [34], the proposal in [35] features an embedded addressing scheme.

The last type of wake-up systems employs ultrasonic communications between receiver and transmitter [36]. The receiver requires less than 1 μA when in idle state and up to 7 μA when active. The system may be employed to estimate distances up to 9 m between receiver and transmitter with a maximum error of 0.1 m. The WuTx consumes 37 mW at 2 V and takes about 0.5 s to specify an 8-bit address, thus its bit-rate can be estimated to be 16 bps.

Optical and ultrasonic WuR systems clearly target different application scenarios than SCM-WuR. However, they can still be compared in terms of several metrics. In fact, the optical approach in [35] outperforms the one in [34] and features the lowest power consumption among all the wake-up systems by only requiring 695 pW in sleeping mode. In turn, the related WuTx only consumes 3 mW to reach up to 50 m. Unfortuntately, the number of applications which can benefit from two perfectly aligned nodes, as required by the optical system since it employs a laser as WuTx, is very restricted compared to SCM-WuR systems, which are not alignment-dependent. On the other hand, while the ultrasonic WuRx in [36] requires less than 1 μA to operate, its application areas are restricted to short-range distance estimation. In addition, its bit-rate is extremely low, even for wake-up applications, making WuC last for up to 500 ms.

### 5.7. Summary Table

The most relevant WuRx proposals in the literature are summarized in Table 3 to provide a way to quickly identify their features, as well as their differences. There are no standardization efforts for WuR systems, thus most of them operate on unlicensed Industrial, Scientific and Medical (ISM) frequency bands. Due to missing information about RFID-WuR proposal in [14], values from the core RFID active tag employed in the WuRx design are indicated. The column labeled as @ indicates if the WuRx features embedded addressing capabilities.

## 6. Conclusions and Future Work

Wake-up Radio (WuR) systems provide significant energy savings for wireless sensors when compared to conventional duty-cycling approaches. In this paper, we investigated and characterized a promising novel WuR approach that is based on SubCarrier Modulation (SCM), which enables two radio operation modes. When the remote sensor node is in low-power *wake-up mode*, a Wake-up Call (WuC) can trigger it remotely. Afterwards, the node switches to *data communication mode* to start the data exchange, e.g., wirelessly reply a transducer measure back.

In this paper, we analyzed the performance results of the SCM-WuR approach and compared them to state-of-the-art WuR systems as of 2013. We conveyed the detailed performance analysis of the SCM-WuR approach in terms of wake-up delay, current consumption and overall operational range. Through physical tests, measurements and simulations, we have shown that SCM-WuR systems feature an outstanding tradeoff between hardware complexity, current consumption and operational range. We also demonstrated how SCM-WuR systems enable multi-hop wake-up for even longer remote sensor measure collection. For this purpose, we implemented a *wake-up relay node* and characterized its operation through delay and current consumption analysis. Finally, we have presented two real sensor monitoring application cases with SCM-WuR system, one single-hop and one multi-hop scenario, and calculated the lifetime and packet delivery ratio of the latter through simulations. Indeed, SCM-WuR systems effectively enable a vast range of applications, while most state-of-the-art proposals are restricted to short and medium-range scenarios. To the best of authors' knowledge, this study is the first one to perform such type of global analysis in the field of Wake-up Radio systems.

As future work, we target the evaluation of SCM-WuR design for different frequency bands, a feature that allows its integration into different wireless technologies.

## Figures and Tables

**Table 1.** Parameters for simulation of operational distances of the SCM-WuR system.

Frequency	868 MHz
WuTx Power	+10 dBm
WuRx Sensitivity	−53 dBm
WuTx Antenna gain	+2 dBi
WuRx Antenna gain	+2 dBi
WuTx Height	1 m
WuRx Height	1.25 m

**Table 2.** SCM-WuR OMNET++ simulation parameter settings.

Frequency	868 MHz
Inter-node Distance	100 m
Packet Payload	100 bytes
Battery Capacity	1,500 mAh
Sleep Current	3.5 μA
Rx Current: Data Communications Mode	18.8 mA
Rx Current: Wake-up Mode	8 μA
Tx Current: Data Communications Mode	17.4 mA
Tx Current: Wake-up Mode (+20 dBm)	152 mA
Data Communications Radio Bit-rate	250 kbps
WuC duration	12 ms

**Table 3.** Comparative of representative WuR proposals in literature.

	**Type**	**Frequency**	**Sensitivity**	**Current Consumption**	**@**	**Range**	**Bit-rate**	**Application Areas**
[1]	RFID	900 MHz (ASK)	−80 dBm	0.2 μA (3 V)	Y	5 m (N/A)	N/A	Short-Range Sensors WBAN
[14]		2.4 GHz (ASK)	−95 dBm	6 μA (3 V)	Y	30 m (0 dBm)	250 kbps	Proof-of-concept

[19]	Heterodyne	2 GHz (OOK)	−72 dBm	104 μA (0.5 V)	N	N/A	200 kbps	WSN
[20]		2.4 GHz (PPM)	−82 dBm	346 μA (1.2 V)	N	N/A	500 kpbs	WSN
[21]		45 MHz (FSK)	−62 dBm	54 μA (0.7 V)	N	<10 m (N/A)	200 kbps	WBAN

[22]	MCU	868 MHz (OOK)	−51 dBm	266.6 μA (3 V)	N	3 m (+4.7 dBm)	N/A	Proof-of-concept

[23]	Low-complexity	433 MHz	N/A	100 μA (1.5 V)	Y	7 m (+10 dBm)	N/A	Proof-of-concept
[6,24]		868 MHz (OOK)	−77 dBm	0.876 μA (3 V)	N	15 m (+27 dBm)	2 kbps	Short-Range Sensor Data-mule WBAN
[25]		2.4 GHz (OOK) 915 MHz (OOK)	−69 dBm −80 dBm	51 μA (1 V)	N	N/A	10 kbps	WSN
[26]		928 MHz (OOK)	−73 dBm	idle/decoding 4.7 μA/599 μA (1.8 V)	Y	N/A	1 kbps	WSN

[5]	Correlator	150 kHz (ASK)	−67 dBm	idle/decoding 2.6 μA/8.3 μA (3 V)	Y	5 m (+33 dBm)	0.5 to 8 kbps	Short-Range Sensors WBAN Proximity keylock
[27]		433 MHz (PWM)	−51 dBm	180 nA (1.5 V)	Y	10 m (+10 dBm)	2 to 80 kbps	Data-mule WBAN Warehouse
[30]		2.4 GHz (PWM)	−50 dBm	19 μA (1 V)	Y	4 m (0 dBm)	50 kbps	Proof-of-concept
[32]		2.4 GHz (OOK)	−55 dBm	8.5 μA (1.5 V)	Y	N/A	100 kbps	Short-Range
[33]		868 MHz (OOK)	−71 dBm	2.4 μA (1 V)	Y	304 m (+6.4 dBm)	20 to 200 kbps	Data-mule Warehouse Environmental

[34]	Optical	Light	−53 dBm	25 μA (3.3 V)	Y	15 m (+12 dBm)	2 kpbs	WSN
[35]		Light (PWM)	37 lux	580 pA (1.2 V)	Y	20 cm (0.5 W LED) 6 m (3 W focus LED) 50 m (3 mW Laser)	91 bps	Proof-of-concept

[36]	Ultrasonic	Sound (OOK)	1 mV at 1 m	874 nA (2 V)	Y	9 m (+15.6 dBm)	16 bps	Distance measurement

SCM	Correlator	868 MHz (OOK)	−53 dBm	idle/decoding 2.7 μA/8.4 μA (3 V)	Y	40 m (+11 dBm) 100 m (+20 dBm)	0.5 to 8 kbps	Short-Range Sensors Data-mule WBAN Warehouse Environmental WSN
